# Atlantic Bluefin Tuna (*Thunnus thynnus*) Biometrics and Condition

**DOI:** 10.1371/journal.pone.0141478

**Published:** 2015-10-27

**Authors:** Enrique Rodriguez-Marin, Mauricio Ortiz, José María Ortiz de Urbina, Pablo Quelle, John Walter, Noureddine Abid, Piero Addis, Enrique Alot, Irene Andrushchenko, Simeon Deguara, Antonio Di Natale, Mark Gatt, Walter Golet, Saadet Karakulak, Ai Kimoto, David Macias, Samar Saber, Miguel Neves Santos, Rafik Zarrad

**Affiliations:** 1 Instituto Español de Oceanografía, C.O. Santander, Santander, Spain; 2 International Commission for the Conservation of Atlantic Tunas, Secretariat, Madrid, Spain; 3 Instituto Español de Oceanografía, C.O. Málaga, Málaga, Spain; 4 National Marine Fisheries Service, Southeast Fisheries Center, Sustainable Fisheries Division, Miami, Florida, United States of America; 5 Institut National de Recherche Halieutique (INRH), Regional Centre of Tangier, Tangier, Morocco; 6 University of Cagliari, Department of Life Science and Environment, Cagliari, Italy; 7 Fisheries and Oceans Canada, St. Andrews Biological Station, St. Andrews, New Brunswick, Canada; 8 Federation of Maltese Aquaculture Producers, Valletta, Malta; 9 International Commission for the Conservation of Atlantic Tunas, GBYP Program, Madrid, Spain; 10 Fisheries Resource Unit, Department of Fisheries and Aquaculture, Marsa, Malta; 11 Gulf of Maine Research Institute, Portland, United States of America; 12 Istanbul University, Faculty of Fisheries, Istanbul, Turkey; 13 National Research Institute of Far Seas Fisheries, Fisheries Research Agency, Shizuoka, Japan; 14 Universidad de Málaga, Departamento de Biología Animal, Málaga, Spain; 15 Institut National des Sciences et Technologies de la Mer, Mahdia, Tunisia; Technical University of Denmark, DENMARK

## Abstract

The compiled data for this study represents the first Atlantic and Mediterranean-wide effort to pool all available biometric data for Atlantic bluefin tuna (*Thunnus thynnus*) with the collaboration of many countries and scientific groups. Biometric relationships were based on an extensive sampling (over 140,000 fish sampled), covering most of the fishing areas for this species in the North Atlantic Ocean and Mediterranean Sea. Sensitivity analyses were carried out to evaluate the representativeness of sampling and explore the most adequate procedure to fit the weight-length relationship (WLR). The selected model for the WLRs by stock included standardized data series (common measurement types) weighted by the inverse variability. There was little difference between annual stock-specific round weight-straight fork length relationships, with an overall difference of 6% in weight. The predicted weight by month was estimated as an additional component in the exponent of the weight-length function. The analyses of monthly variations of fish condition by stock, maturity state and geographic area reflect annual cycles of spawning and feeding behavior. We update and improve upon the biometric relationships for bluefin currently used by the International Commission for the Conservation of Atlantic Tunas, by incorporating substantially larger datasets than ever previously compiled, providing complete documentation of sources and employing robust statistical fitting. WLRs and other conversion factors estimated in this study differ from the ones used in previous bluefin stock assessments.

## Introduction

Fish size is biologically relevant because several ecological and physiological factors are size-dependent. Consequently, variability in size has important implications for diverse aspects of fisheries science and population dynamics [1]. Size-based analyses of marine animals are becoming increasingly popular methods for improving the understand of community structure and function [2], while weight-length relationships have several diverse applications, namely in fish biology, physiology, ecology, and fisheries assessment [3]. Weight-length, length-length and weight-weight relationships are important in fisheries science, notably to raise length-frequency samples to total catch, to estimate biomass from length observations, to convert one type of length to another, or to calculate fish condition. Furthermore weight-length relationships have often been used to estimate weight from length, as direct weight measurements in the field can be time-consuming or simply cannot be taken due to dockside processing practices. Weight-length relationships are required to obtain conversion factors for use by the International Commission for the Conservation of Atlantic Tunas (ICCAT) dealing with different types of data.

Atlantic bluefin tuna (*Thunnus thynnus* [L. 1758]) is a highly migratory species, commonly distributed throughout the North Atlantic and Mediterranean Sea in subtropical and temperate waters [4]. The ICCAT has defined two separate stocks, Eastern (including the Mediterranean Sea) and Western Atlantic divided by the 45°W meridian, based on different biological features such as homing behavior, spawning site fidelity, genetic differentiation and differing ages for reaching sexual maturity [5–9]. These biological differences include seasonal differences in somatic condition, mainly due to the dissimilar timing of spawning. All of these factors make it advisable to evaluate biometrics of this species by stock.

The current conversion factors for this species were obtained more than thirty years ago and some of them are not well documented. In addition, some of the current ICCAT conversion factors have been called into question for both Atlantic bluefin stocks [10–12]. Furthermore, interest in the biometric relationships of this species has increased in recent years due to their use as a means of determining the growth of fish fattened in cages in the Mediterranean [13]. Although a number of biometric relationships for bluefin have been regularly submitted to ICCAT, most are based on samples from different fractions of the population, partly because they were obtained from sampling seasonal fisheries in a reduced geographical area and/or sampling of a limited size range due to gear selectivity and management regulations. These limitations make it difficult to obtain representative samples for developing meristic relationships. To ensure broad spatial, temporal, size range and fishing gear coverage, several scientific institutions and fishing organizations have combined their databases to provide new and updated bluefin biometric relationships for all of the distribution areas, months and several years in the North Atlantic Ocean and Mediterranean Sea. Thus, the main objectives of this study were to obtain robust estimations of biometric relationships for Atlantic bluefin tuna by stock, perform sensitivity analyses to evaluate the representativeness of sampling and explore the procedures to fit the weight-length relationship. Furthermore, seasonal variability in somatic condition (weight-length relationship) for bluefin was investigated in relation to reproductive status and geographical location, in order to explore whether condition variability reflects seasonal changes associated to spawning costs and feeding.

## Material and Methods

### Sampling

**Fig 1:**
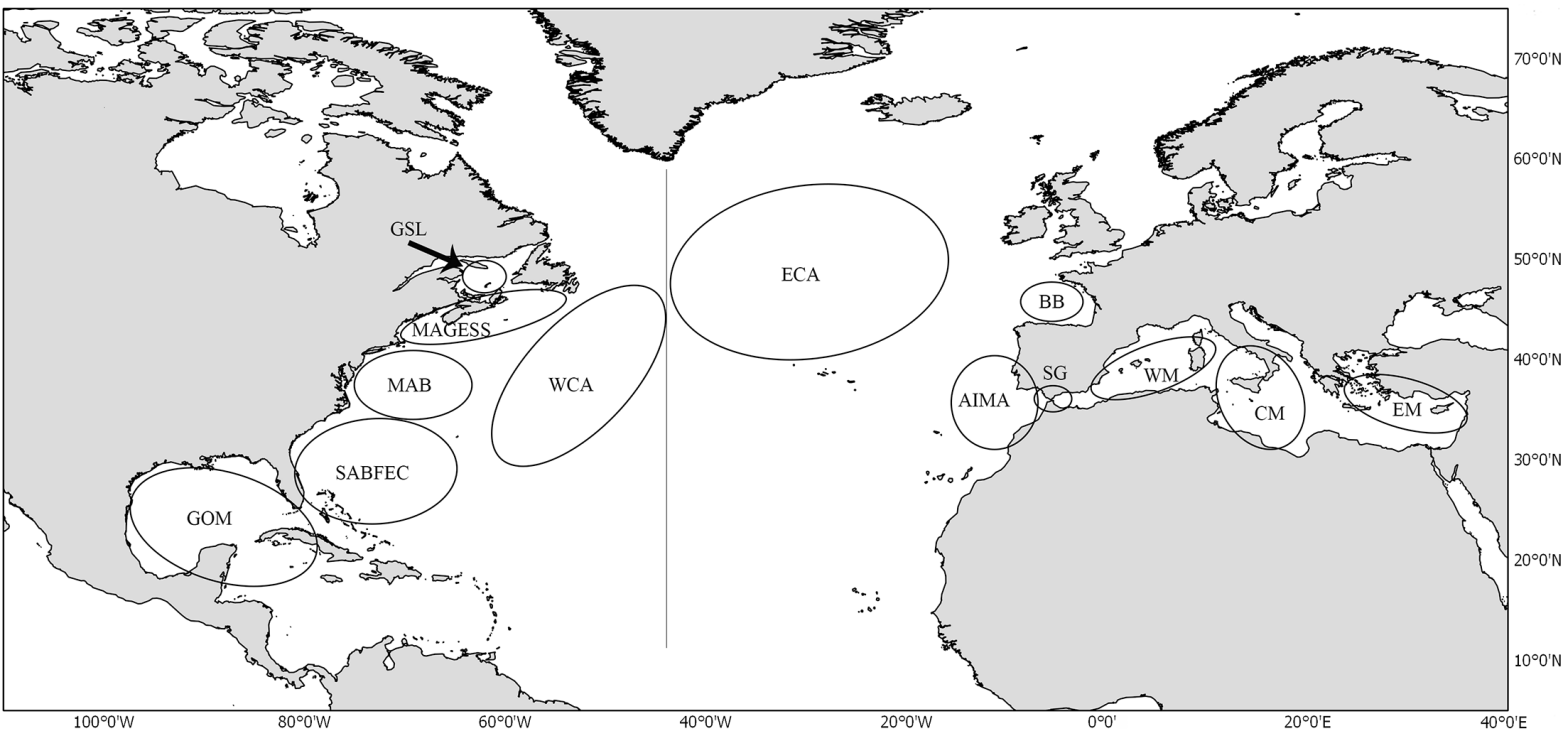
Sampled geographic areas. GOM = Gulf of Mexico, SABFEC = South Atlantic bight, Florida East Coast and Sargasso Sea, MAB = Mid-Atlantic Bight, MAGESS = Gulf of Maine, George’s Bank and Scotian Shelf, GSL = Gulf of St. Lawrence, WCA = Western Central Atlantic, ECA = Eastern Central Atlantic, BB = Bay of Biscay, AIMA = Atlantic Iberian-Moroccan Area, SG = Strait of Gibraltar, WM = Western Mediterranean, CM = Central Mediterranean, and EM = Eastern Mediterranean.

Biometric relationships were based on the sampling of Atlantic bluefin tuna landings by different fishing gears for the time period from 1998 to 2012 (Table 1). Catch locations were grouped into several geographic areas (Fig 1): 1- Gulf of Mexico (GOM); 2- South Atlantic bight, Florida East Coast and Sargasso Sea (SABFEC); 3- Mid-Atlantic Bight (MAB); 4- Gulf of Maine, George’s Bank and Scotian Shelf (MAGESS); 5- Gulf of St. Lawrence southern waters (GSL); 6- Western Central Atlantic (WCA) delimited by 25 to 50°N and 45 to 80°W; 7- Eastern Central Atlantic (ECA), delimited by 45 to 60°N and 15 to 45 °W; 8- Bay of Biscay (BB); 9- Atlantic Iberian-Moroccan Area (AIMA), delimited longitudinally by the Atlantic area from 15°W to 7°W and latitudinally from the Algarve coast (southern Portugal) to the Moroccan coast; 10- Strait of Gibraltar (SG) defined as the area between 7–5°W; 11- Western Mediterranean (WM), comprising the Alboran Sea and waters around the Balearic Islands and Sardinia; 12- Central Mediterranean (CM), including the Ionian Sea, Tyrrhenian Sea and the Strait of Sicily and 13- Eastern Mediterranean (EM), comprising Aegean Sea and Northern Levantine Sea.

**Table 1:** Number of sampled Atlantic bluefin tuna and month of capture by geographical zone and fishing gear. Geographical areas: BB = Bay of Biscay, AIMA = Atlantic Iberian-Moroccan Area, SG = Strait of Gibraltar, ECA = Eastern Central Atlantic, WCA = Western Central Atlantic Central, GOM = Gulf of Mexico, GSL = Gulf of St. Lawrence, MAB = Mid-Atlantic Bight, MAGESS = Gulf of Maine, George’s Bank and Scotian Shelf, SABFEC = South Atlantic bight, Florida East Coast and Sargasso Sea, WATL = Unidentified western Atlantic, EM = Eastern Mediterranean, CM = Central Mediterranean, WM = Western Mediterranean

Geographic area	Bait boat	Gillnet	Handline	Harpoon	Longline	Purse seine	Tended line	Trap	Rod & Reel and Troll	Unknown	Total
GOM					301 (Jan-Dec)						301
SABFEC			2782 (Jan-Dec)	4 (Nov-Dec)	215 (Jan-Dec)					1 (Feb)	3002
MAB			2311 (Jan-Dec)	15 (Jun-Oct)	287 (Jan-Dec)	89 (Oct)				1 (Jun)	2703
MAGESS			20804 (Jan-Dec)	4222 (Jun-Nov)	325 (Apr-Dec)	5601 (Jul- Oct)	945 (Jul-Oct)		5251 (May-Nov)	3 (Jul-Nov)	37151
GSL				7 (Aug-Oct)			119 (Jul-Oct)		7492 (Jul-Nov)	41 (Jul-Nov)	7659
WCA					3234 (Jan-Dec)						3234
WATL			15 (Jul-Nov)						19 (Jul-Oct)		34
Total WAtl			25912 (Jan-Dec)	4248 (Jun-Dec)	4362 (Jan-Dec)	5690 (Jul-Oct)	1064 (Jul-Oct)		12762 (May-Nov)	46 (Feb-Nov)	54084
ECA					9935 (Jan-Dec)						9935
BB	358 (Jun-Oct)								2 (Aug)		360
AIMA					88 (Jan-Dec)			993 (Apr-Oct)			1081
SG	47411 (Jan-Dec)		2389 (May-Sep)		22 (Jan-Nov)			1436 (Apr-Jul)	1 (Jul)		51259
Total EAtl	47769 (Jan-Dec)		2389 (May-Sep)		10045 (Jan-Dec)			2429 (Apr-Oct)	3 (Jul-Aug)		62635
WM	2 (Jul)	39 (Feb)			6832 (Jan-Dec)	60 (Jun-Jul)			188 (Jul-Nov)		7121
CM		4384 (Jun-Oct)	38 (Apr-Dec)		8664 (Jan-Dec)	1192 (May-Nov)		1640 (May-Jun)			15918
EM						2361 (Jan-Dec)					2361
Total Med	2 (Jul)	4423 (Jun-Oct)	38 (Apr-Dec)		15496 (Jan-Dec)	3613 (Jan-Dec)		1640 (May-Jun)	188 (Jul-Nov)		25400
Total	47771 (Jan-Dec)	4423 (Jun-Oct)	28339 (Jan-Dec)	4248 (Jun-Dec)	29903 (Jan-Dec)	9303 (Jan-Dec)	1064 (Jul-Oct)	4069 (Apr-Oct)	12953 (May-Nov)	46 (Feb-Nov)	142119

**Fig 2:**
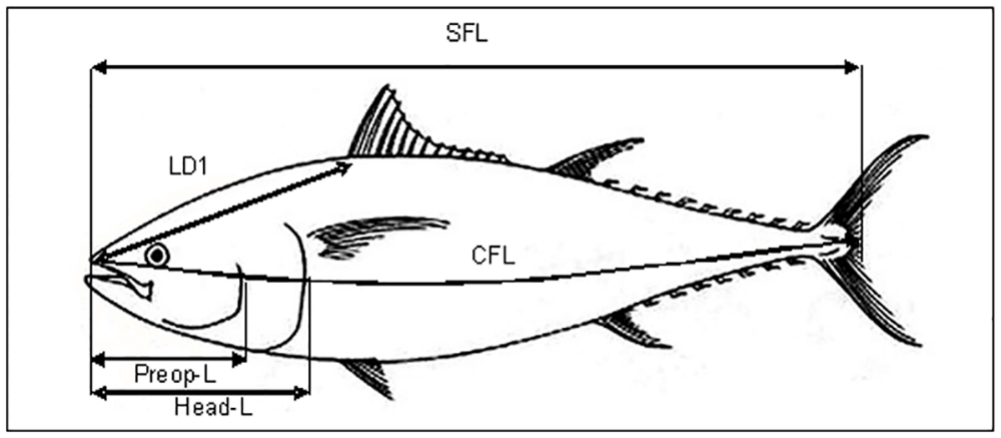
Types of measurements of Atlantic bluefin tuna used in the present study. Straight fork length (SFL), Straight first dorsal fin length (LD1), Curved fork length (CFL), Head length (HeadL) and Preopercular length (PreopL).

Bluefin tuna specimens were measured and weighed when they were dead and after being caught by the various fishing gears. Most of the sizes and weights were taken from tuna landed in ports and on board fishing vessels, but also a small percentage of the sampling was done in research laboratories. All bluefin tuna caught and used in this study come from the allowable catch of the respective countries. These official catches comply with the total allowable catches and quotas allocated by ICCAT. Length and weight measurements were obtained and recorded directly with no conversions. Measurement units were all standardized to centimetres for length and kilograms for weight. Different length measurements were collected by the different sources of data of this study (Fig 2), including: Straight fork length (SFL): the straight line from the tip of the upper jaw to the posterior of the shortest caudal ray (fork of the caudal fin); Curved fork length (CFL): the length from the tip of the upper jaw to the fork over the fish curvature body; Straight first dorsal fin length (LD1): the straight line from the tip of the upper jaw to the base of the first dorsal spine (the start of the first dorsal fin); Head length (HeadL): the straight line from the tip of the upper jaw to the posterior border of the operculum; and Preopercular length (PreopL): this is the straight line from the tip of the upper jaw to the posterior border of the preoperculum. The following measurements of weight were used: Round weight (RWT): the weight of the whole fish as it comes out of the water before any processing or dressing; Gutted weight (GWT): the weight of the fish without guts and gonads, but with head, tail (caudal fin) and gills; Gutted and gilled weight (GGWT): the weight of the fish without guts, gills and gonads, but with head and tail; Gutted, gilled and tailed weight (GGTWT): the weight of the fish without guts, gills, gonads and tail, but with head; and Dressed Weight (DWT): the weight of the fish gutted, head off and tail off.

### Data fitting and sensitivity analysis

Estimation of biometric relationships (weight-length, length-length and weight-weight, WLR, LLR and WWR, respectively) was restricted to variables with good sampling coverage for the entire size range, representing most of the season and geographical area strata. LLR and WWR were estimated by robust linear fitting [14], while WLR were estimated through Gauss-Newton nonlinear regression fitting. Outliers were excluded only when detected as obvious errors, while the influence of other potential remaining outliers was minimized using robust regression fitting procedures [14].

The majority of the samples were obtained from fisheries dependent programs targeting different fractions of the population, with varying seasonal patterns, using several types of fishing gears, and measured using a variety of measurement types. Therefore, despite the extensive dataset assembled from several areas throughout the Atlantic Ocean and the Mediterranean Sea, size-weight sampling was unbalanced. To maximize the spatial, geographical and size/weight coverage, different types of size and weight measurements were standardized to common units of size (straight fork length, SFL_std) and weight (round weight, RWT_std). These provided a wider size range for both stocks, greatly expanding the spatial, temporal and fishing gear coverage of sampling for the Eastern stock in the Mediterranean (where the majority of catches are occurring), while improving Western stock length range sampling by including very large fish mainly present in Canadian and USA fisheries (S1 Fig). Standardization was performed using the robust fitting relationships for those LLR and WWR that had very high coefficients of determination (*r*-square ≥ 0.98): east Atlantic LLR: CFL to SFL and WWRs: GWT and GGWT to RWT; west Atlantic LLR: CFL to SFL and WWR: DWT to RWT.

In addition, simulation analyses were conducted to evaluate the representativeness of the sampling for each stock. The base simulations estimated the expected size sampling distribution of a bluefin-like population if it were randomly sampled using a completely non-selective gear. A population model was constructed using the bluefin biological parameters of natural mortality, age and size-at-age used in Virtual Population Analysis models [15], and assuming a variance of size at age equivalent to a coefficient of variation (CV) of 12.5% for all ages. Then, taking into account that the numbers of fish in each cohort decreases with age due to natural and fishing mortality, the expected sampling per 1 cm bin size (SFL) proportions were estimated for both stocks assuming a non-selective gear. Since most of the sampling comes from fishery dependent sources where both selectivity and management regulations prohibit the retention of small fish, an assumed knife edge-type selectivity pattern of 170 cm SFL and 75 cm SFL for Western and Eastern stock (respectively) was introduced in the simulation population model to account for ICCAT and U.S. management regulations related to minimum size (U.S. commercial fisheries represent 65% of the Western size data and their national regulations impose minimum size retention of 185 cm CFL).

**Fig 3:**
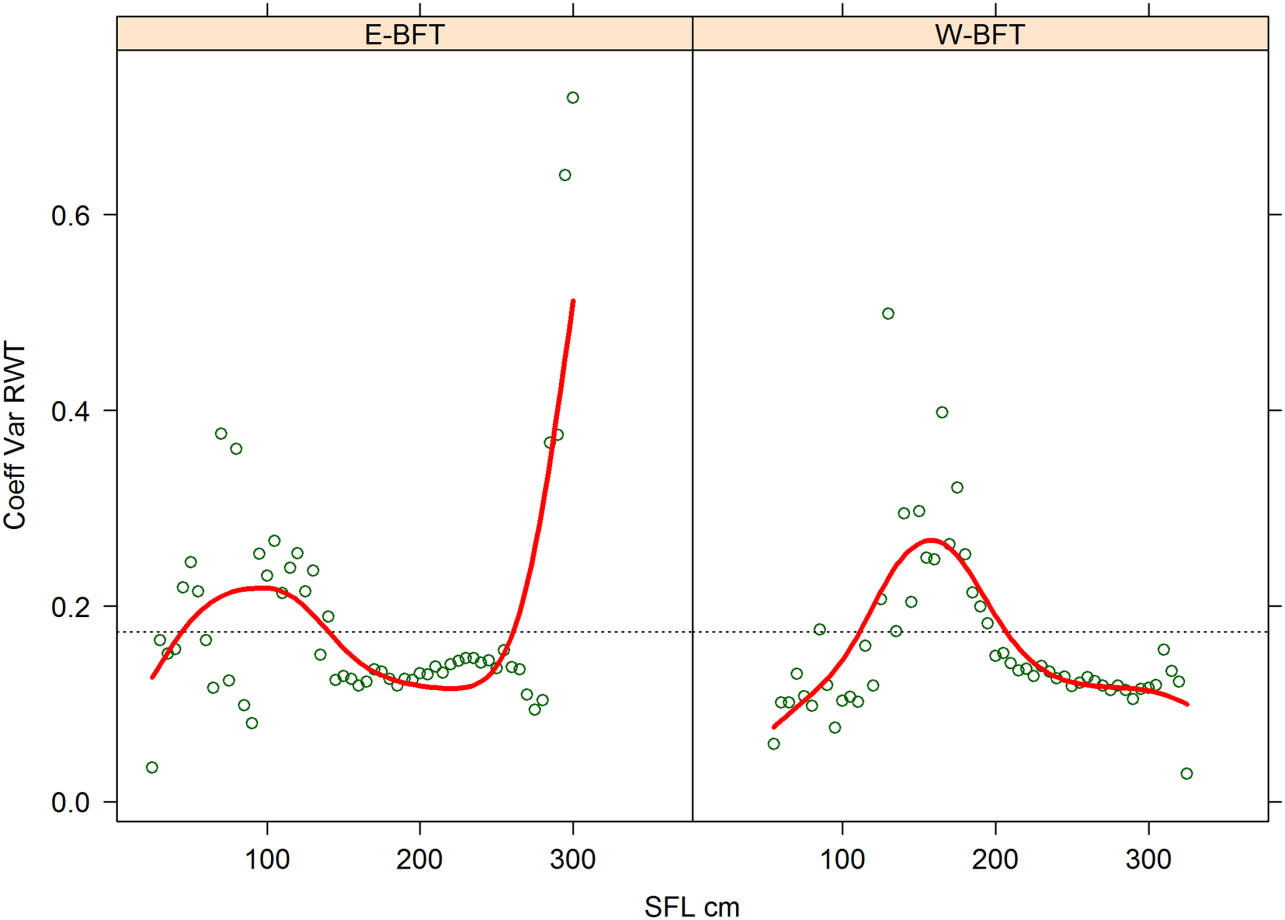
Coefficient of variation (CV) of weight at size for 5 cm SFL bins. Eastern (left panel) and Western (right panel) Atlantic bluefin tuna (BFT).

Additional analyses were performed to identify robust procedures to fit the RWT-SFL relationship. Traditional allometric scaling laws for relationships between biological parameters like volume and length (e.g., weight and length) are well-known examples of power-law functions. However, the mean is usually well defined for these types of functions when the exponent is greater than 2, but is only true in the case of variance when the exponent exceeds 3 [16]. This poses problems when applying traditional statistics based on variance or standard deviation, such as regression analysis, or when estimating confidence intervals for predicted values [16]. In general it is expected that with allometric scaling, the variance of the dependent variable (e.g., weight) increases continuously along with the independent variable. Analyses of standardized input data showed a trend of variance for weight at size (Fig 3). For small Eastern bluefin (< 60 SFL) CV of weight at size ranged from 15% to 25%; between 60 to 150 cm SFL the CV decreased to between 6 to 12.5%; while larger than 150 cm SFL the overall CV remained about 12.5%. For Western bluefin smaller than 120 cm SFL, the CV of weight at size ranged between 8 to 18%; then the CV increased between 120 and 200 cm SFL, ranging from 20% to 50%; while larger than 200 cm SFL the CV average decreased to about 12.5%. However for fish closer to 300 cm SFL, an extremely large variance of weight at size was observed. Because of these trends, a weighted nonlinear regression was employed to reduce the influence of observations with larger than expected variance of weight at size, by using the inverse of the estimated CV of weight at 5 cm length bins as the weighting factor.

To evaluate the influence of larger variance of weight at size and to obtain more realistic confidence bounds for the predicted mean weight at size, two other fitting approaches were considered: 1) nonlinear fitting using quantile regression and 2) bootstrap analyses. In theory, the curves obtained through quantile regression offer distribution of weights at given lengths and are less sensitive to extreme values [17]. Quantile regression was estimated using the R package quantreg [18].

Nonparametric weighted bootstrap analyses were performed to address two separate issues: a) the influence of observations with larger variance of weight at size and how it affected the estimation of alpha and beta parameters, and b) the effect of non-uniform number of samples per size classes (5 cm SFL intervals). In case a) the bootstrap procedure allocated the same number of observations with equal resampling probability for all data by size bin. In case b) the bootstrap scheme was modified to produce a sample bootstrap with a constant number of samples for each 5 cm size class bin within the range of 30 to 300 cm. This second approach achieves effectively a scenario of “*what if*” an equal number of observations were available for each 5 cm size class. Bootstrap analyses were conducted using the R boot package [19, 20] and a non-linear least squares fitting platform [21]. A total of 1000 bootstrapped replicates were produced and distributions of the parameters alpha, beta, and residual square mean error were evaluated.

Finally, seasonal effects were investigated to evaluate changes in weight related to reproductive status (mature versus immature) and by geographical area. Annual variability was also explored. The approach was to use a GLM model where the dependent variable was the scaled residuals (obs-Pred)/Pred from the final fitted weight-size model (non-linear weighted by the inverse of the CV model), and the predictor variables considered were size SFL_std as a continuous predictor, with month, maturity and main geographical areas as fixed factors. Scaled residuals were used because raw residuals increase in absolute value with fish size. Scaled residuals with values greater than 1 were excluded, as these indicate that the observed weight was more than double the weight predicted by the model; overall less than 0.3% of observations were excluded by stock. These analyses were initially performed by stock and by month, though further exploration was done within geographic and maturity status strata when possible. For the latter, it was assumed that Western bluefin tuna mature at SFL greater or equal to185 cm (L_100%mat_ [22]), while for Eastern bluefin it was considered that maturity was reached at SFL greater or equal to 130 cm (L_100%mat_ [23]). For Eastern bluefin, 3 major geographical areas were defined: the east Atlantic; the western and central Mediterranean Sea (including the Strait of Gibraltar area); and the eastern Mediterranean. Nevertheless, geographical areas were not equally sampled, with west-central Mediterranean representing more than 90% of the data. For Western bluefin, the Gulf of Mexico was considered the main spawning area [24]; however the limited number of observations (< 1000) precluded modeling of this as an independent area factor.

The estimated weight-length equation including the monthly factor as an indicator of fish condition was estimated as:

$$RWT\_std_{M}\;=\;alpha\times SFL\_std^{\left(beta+(LsMean_{M}\times MeanSquareError\right))}$$

where M = 1, …12 (month)

Monthly least square means (LSMeans) were used to investigate departure patterns in expected weight at size (as a proxy of fish condition) by month compared to the annual average. Least square means were used because of the imbalance in the number of observations per month [25].

## Results

Nearly all of the biometric length-length and weight-weight relationships showed high values of coefficients of determination (*r*-square) (Tables 2 and 3). These functions were used to convert all data to common standard units of round weight (RWT in kg) and straight fork length (SFL in cm) to fit the weight at length relationship by stock.

**Table 2:** Atlantic bluefin tuna biometric relationships for the Eastern Atlantic and Mediterranean stock. Independent and dependent variables (X and Y), number of specimens (n), parameters of the linear and nonlinear equations and coefficient of determination (r²). Straight fork length (SFL), curved fork length (CFL), straight first dorsal fin length (LD1), head length (HeadL), preopercular length (PreopL), round weight (RWT), gutted weight (GWT), gutted and gilled weight (GGWT), gutted, gilled and tailed weight (GGTWT) and dressed weight (DWT). Method A: Fit Robust Estimate; B: Nonlinear fit CV weighted; C: Nonlinear fit Gauss-Newton. Standardized WLRs (RWT_std-SFL_std). Length in centimeters and weight in kilograms.

East and Mediterranenn stock unit	X	Y	X range	Y range	n	Months sampled	alpha	beta	r²	Residual standard error	Method
**Length conversion factors**:											
LD1 = alpha + beta × SFL	SFL	LD1	56–300	17–71	636	2–8, 10, 11	5.6891	0.2543	0.978	2.052	A
CFL = alpha + beta × SFL	SFL	CFL	78–242	84–252	222	6–7	-1.887	1.0507	0.990	4.121	A
SFL = alpha + beta × LD1	LD1	SFL	17–71	56–300	636	2–8, 10, 11	-19.733	3.8648	0.978	8.063	A
CFL = alpha + beta × LD1	LD1	CFL	24–71	84–283	312	5–7	-27.832	4.1273	0.964	8.839	A
LD1 = alpha + beta × CFL	CFL	LD1	84–283	24–71	312	5–7	7.9182	0.2355	0.964	2.116	A
SFL = alpha + beta × CFL	CFL	SFL	84–252	78–242	222	6–7	2.9457	0.9442	0.990	3.886	A
HeadL = alpha + beta × CFL	CFL	HeadL	84–284	22–74	306	5, 7	4.4041	0.2242	0.865	3.048	A
PreOP = alpha + beta × CFL	CFL	PreOP	153–284	33–74	294	5	1.0934	0.1892	0.646	3.100	A
PreOP = alpha + beta × HeadL	HeadL	PreOP	38–74	33–74	294	5	-2.2179	0.8358	0.783	2.428	A
**Weight conversion factors**:											
GWT = alpha + beta × RWT	RWT	GWT	0.3–370	0.3–358	236	5–11	-0.2169	0.9540	1.000	1.090	A
GGWT = alpha + beta × RWT	RWT	GGWT	3–300	2.8–239	187	5–8, 10	1.2985	0.7421	0.991	5.918	A
RWT = alpha + beta × GGWT	GGWT	RWT	2.8–239	3–300	187	5–8, 10	-1.6151	1.3373	0.991	7.812	A
RWT = alpha + beta × GWT	GWT	RWT	0.3–358	0.3–370	236	5–11	0.2312	1.0479	1.000	1.140	A
**Weight—length relations**											
RWT_std = alpha × SFL_std^beta^	SFL	RWT	27–300	0.25–513	74272	1–12	3.51E-05	2.8785	na	15.965	B
GGTWT = alpha × SFL^beta^	SFL	GGTWT	75–281	8–362	8034	1, 8–12	4.59E-05	2.8077	na	13.407	C
GGWT = alpha × SFL^beta^	SFL	GGWT	55–289	2.8–385	3469	1–12	1.07E-04	2.6301	na	14.249	C
GGWT = alpha × CFL^beta^	CFL	GGWT	94–289	10–338	4962	4–8	2.55E-05	2.8938	na	15.357	C
GGWT = alpha × LD1^beta^	LD1	GGWT	29–76	20–350	2044	5–7, 9	3.85E-03	2.6211	na	21.820	C
RWT = alpha × LD1^beta^	LD1	RWT	17–79	3–425	2796	2–8, 10–11	1.12E-03	2.9180	na	20.019	C

**Table 3:** Atlantic bluefin tuna biometric relationships for the Western Atlantic stock. Independent and dependent variables (X and Y), number of specimens (n), parameters of the linear and nonlinear equations and coefficient of determination (r²). Straight fork length (SFL), curved fork length (CFL), straight first dorsal fin length (LD1), head length (HeadL), preopercular length (PreopL), round weight (RWT), gutted weight (GWT), gutted and gilled weight (GGWT), gutted, gilled and tailed weight (GGTWT) and dressed weight (DWT). Method A: Fit Robust Estimate; B: Nonlinear fit CV weighted; C: Nonlinear fit Gauss-Newton. Standardized WLRs (RWT_std-SFL_std). Length in centimeters and weight in kilograms.

West stock unit	X	Y	X range	Y range	n	Months sampled	alpha	beta	r²	Residual standard error	Method
**Length conversion factors**											
SFL = alpha + beta × CFL	CFL	SFL	55–274	53–265	1035	3, 6–10	1.8575	0.9606	0.991	2.565	A
CFL = alpha + beta × SFL	SFL	CFL	53–265	55–274	1035	3; 6–10	-0.8319	1.0314	0.991	2.670	A
**Weight conversion factors**											
RWT = alpha + beta × DWT	DWT	RWT	93–637	70–514	1960	7–10	6.1971	1.2303	0.976	12.581	A
DWT = alpha + beta × RWT	RWT	DWT	70–514	93–637	1960	7–10	0.2911	0.7967	0.976	10.135	A
**Weight—length relations**											
RWT_std = alpha × SFL_std^beta^	SFL	RWT	53–353	4–637	51204	1–12	1.77E-05	3.0013	na	30.651	B
RWT = alpha × CFL^beta^	CFL	RWT	56–338	4–637	2977	3, 6–10	4.94E-05	2.8094	na	32.625	C
DWT = alpha × CFL^beta^	CFL	DWT	127–366	25–514	49344	1–12	8.31E-06	3.0780	na	24.750	C
GGTWT = alpha × SFL^beta^	SFL	GGTWT	92–289	11–403	2324	1–3, 9–12	1.27E-05	3.0491	na	18.242	C

**Fig 4:**
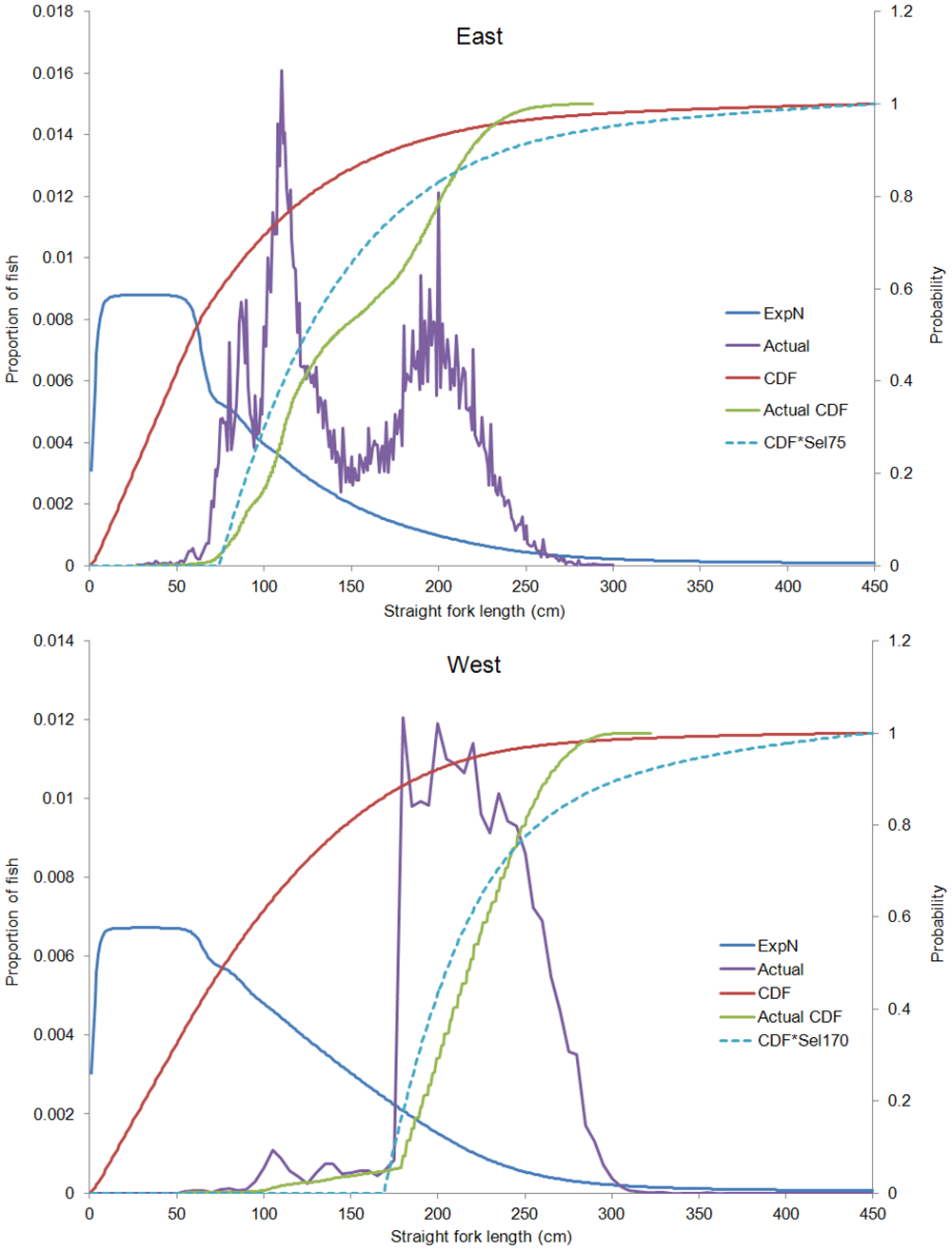
Representativeness of present sampling by stock. Proportion of fish by size expected to be sampled in the case of a population without fishing mortality (ExpN), with the red line representing its cumulative density function (CDF). The actual proportion of size samples (Actual) and its cumulative density function (Actual CDF) are also shown. The dashed blue line represents the simulated population assuming a knife edge selectivity at 75 SFL (CDF*Sel75) for Eastern (top panel) and at 170 SFL (CDF*Sel170) for Western (bottom panel) bluefin tuna stocks.

For the representativeness of the compiled data for each stock, comparison of the observed data versus a simulated bluefin-like population (Fig 4) indicated that for Eastern bluefin most of the samples of a population would be of small fish (60% of fish < 76 cm SFL), while less than 7% of samples would be of fish ≥ 200 cm SFL. Eastern bluefin size samples show a bimodal peak with reduced sampling in the 130–180 cm SFL. Although the size data are clearly missing small fish, with the 75 cm SFL knife edge selectivity (Fig 4 top panel dashed line) the expected and actual sampling proportions are much closer for all sizes except 130–180cm SFL. Sampling of Western bluefin is truncated for fish below 175 cm SFL (Fig 4 bottom panel). Overall there is generally good agreement, but the proportion of larger fish sampled (East ≥ 210 cm SFL and West ≥ 230 cm SFL) is higher than would be expected in a simulated population.

**Fig 5:**
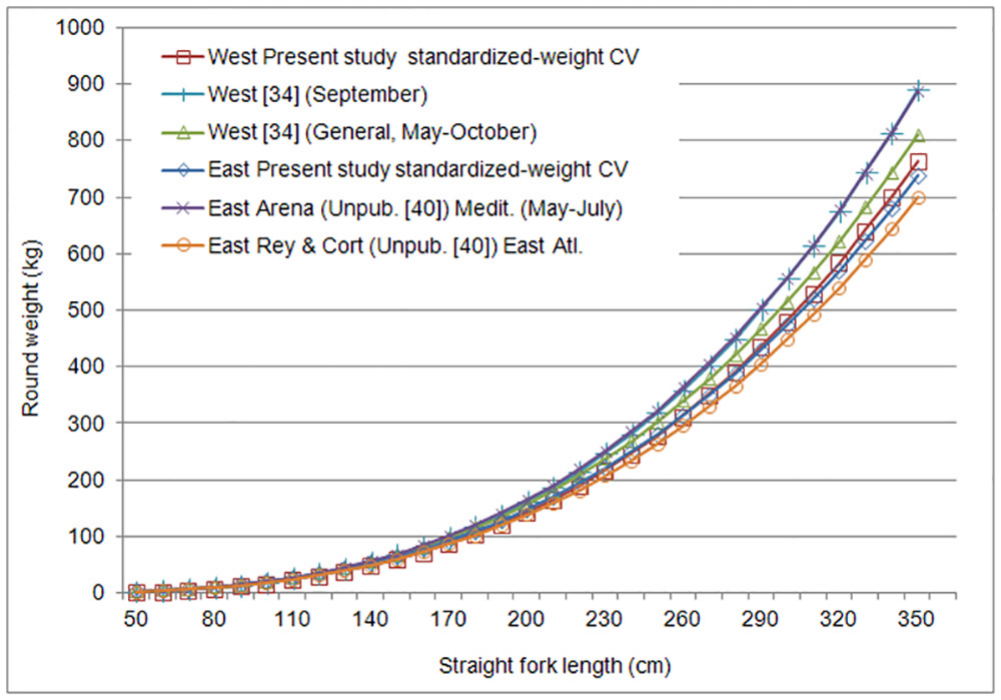
Present study results for the weight-length (RWT-SFL) relationship by stock compared with equations currently used for bluefin tuna. West = West Atlantic, East = East Atlantic, including the Mediterranean.

We modeled WLR by stock using nonlinear regression applied to standardized data (RWT_std—SFL_std) and weighted by the inverse of the CV. Results of these functions are presented in Tables 2 and 3. The estimated annual WLRs were similar for both stocks, with an overall difference of 6% (19 to 11% for SFL cm<110, 10 to 6% for 110>SFL cm<170 and less than 5% for SFL cm>170) (Fig 5).

**Fig 6:**
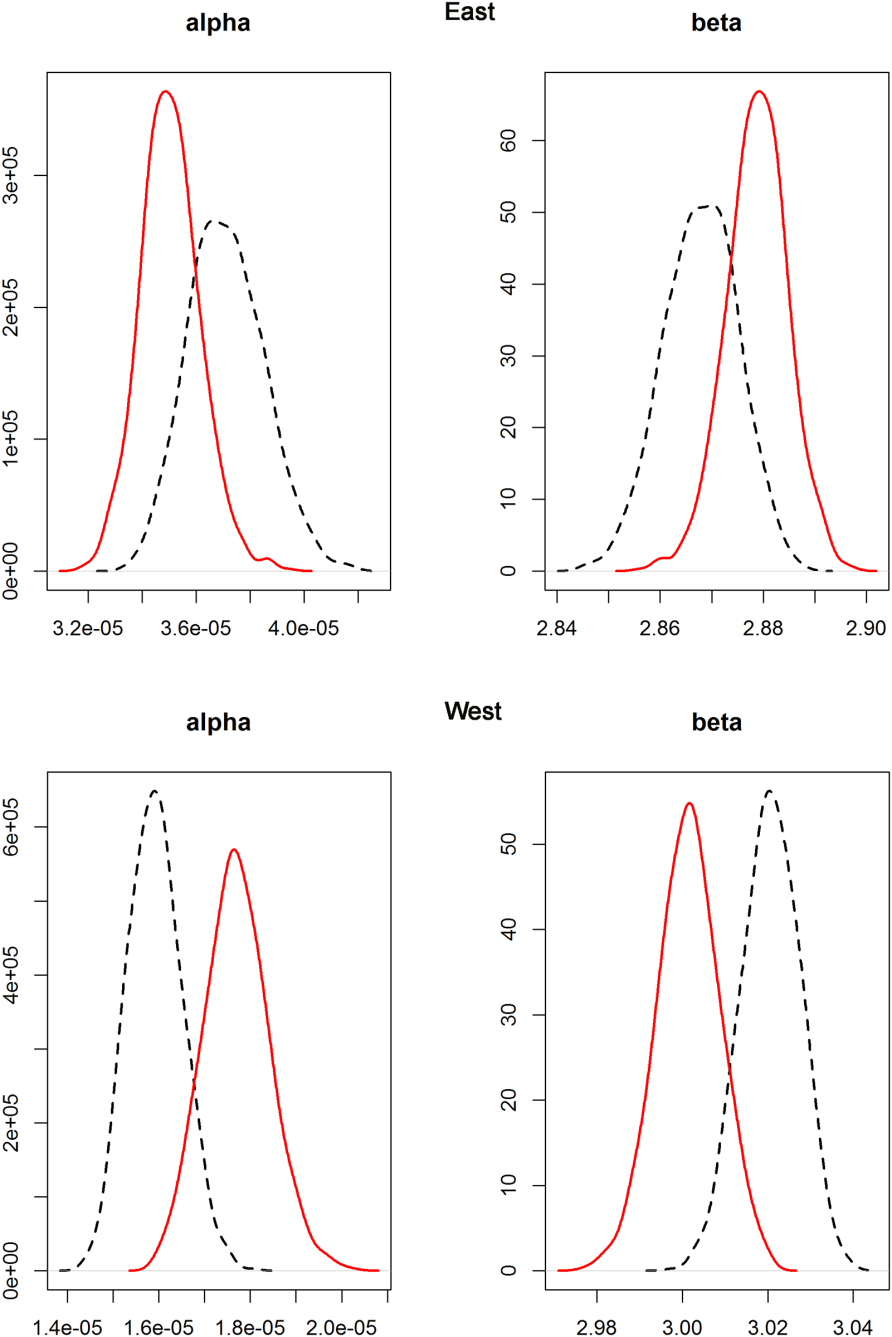
Density distributions of bootstrap estimates of alpha and beta parameters. Un-weighted (dashed line) and weighted (continuous line) models for the Eastern (top panel) and Western (bottom panel) Atlantic bluefin tuna.

The un-weighted and CV-weighted WLRs showed almost no difference for either stock. The density distributions of the alpha and beta parameters from the bootstrapped analyses showed a symmetric distribution (Fig 6), indicating non-significant bias in the parameter estimates and overall robust estimation. Despite the high correlation of the alpha and beta parameters, the model did consistently find an overall minimum solution with the available data. The CV-weighted nonlinear model was chosen to minimize the undue influence of highly variable weight at size observations.

**Fig 7:**
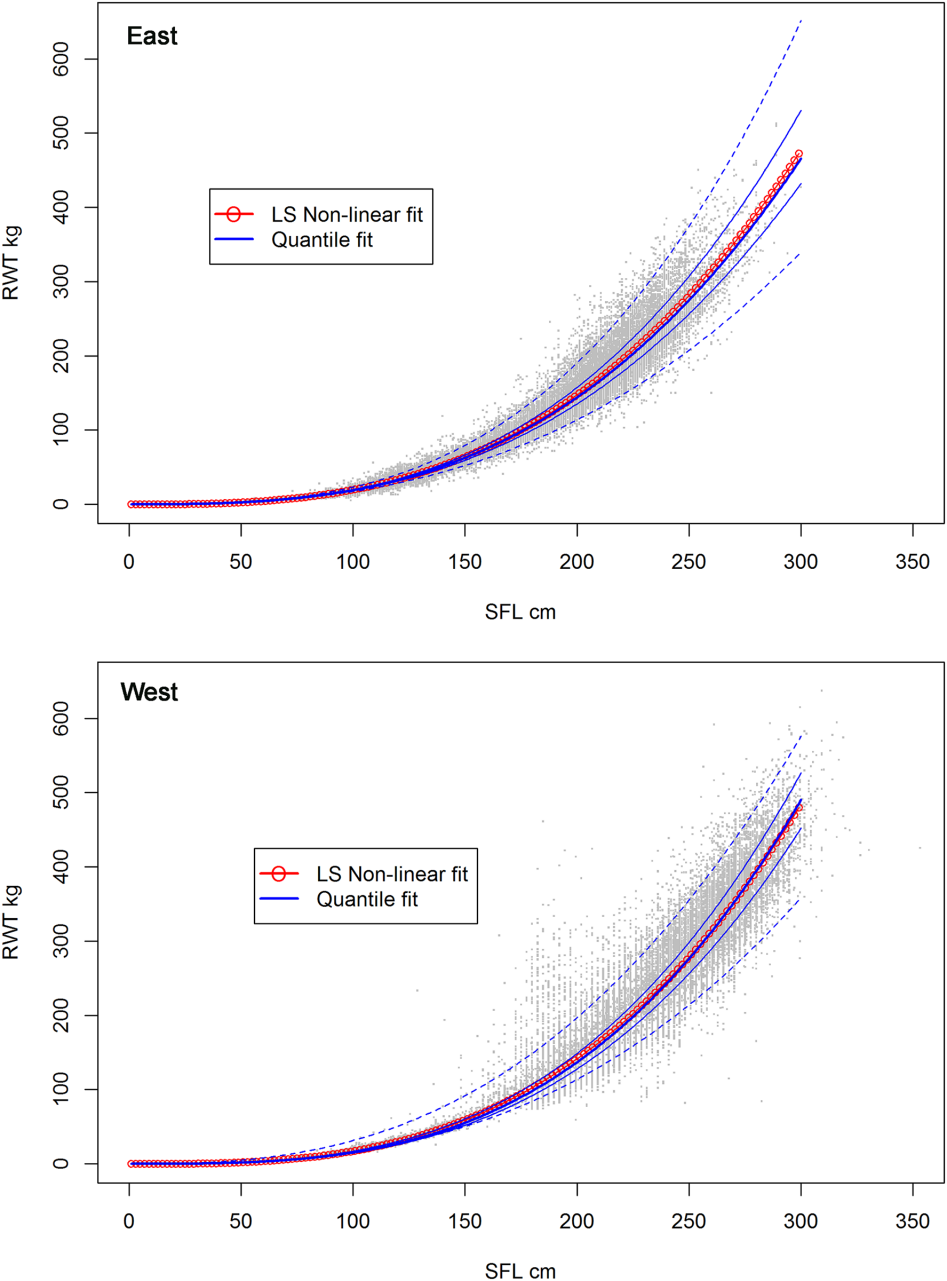
Comparison of quantile regression median and least squares of weight at size. Quantile median non-linear fit (continuous line) and least squares weighted non-linear fit (line with circles) by Eastern (top panel) and Western (bottom panel) bluefin tuna stocks. Outer lines represent the 25%, 75% (thin lines) and the 2.5% and 97.5% (dashed lines) of the quantile regression estimated percentiles.

The comparison of quantile regression median (0.5 quantile) and least squares (LS) showed similar predictions for both stocks (Fig 7). For the Eastern stock, the median quantile regression tended to estimate a slightly lower weight at size compared to the LS fit, but differences in weight were 3% or less for larger fish (SFL > 300 cm). For the Western stock, the quantile regression indicated smaller weight at size from 50 to 270 cm SFL, but higher weight for fish > 270 cm SFL. However, the quantile estimation of confidence bounds, as shown by the 25%– 75% percentile range, widens as size increases for both stocks. The 95% quantile range is much wider, clearly reflecting the large observed variations of weight at size for both stocks. In addition, the quantile regression indicates that estimated variance is not symmetric about the median values, particularly in the case of the Western stock.

The bootstrap analyses also provided alternative confidence intervals for the WLRs. Yet, the 95% quantile range of a thousand bootstraps resulted in approximate upper and lower confidence limits that were so close to the median, with average CVs below 1%, that they could not be visually detected (Fig 7). This is in contrast with the confidence intervals estimated by the quantile regression (Fig 7) which reflected increased variability in weight at higher lengths.

**Fig 8:**
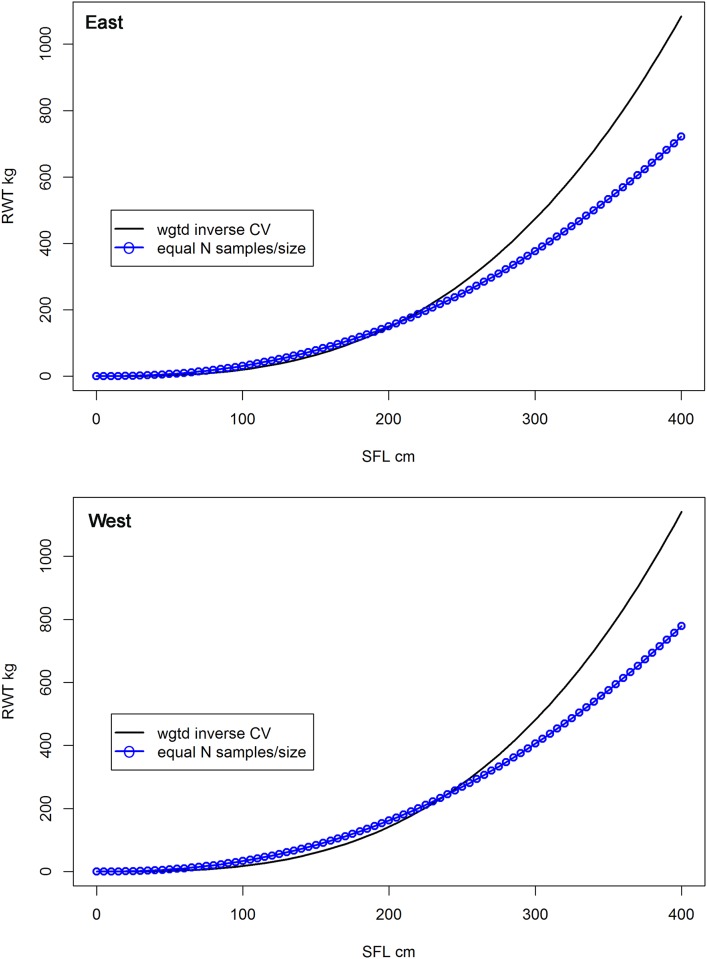
Comparison of the weighted model versus equal number of observations per size class model. Eastern (top panel) and Western (bottom panel) Atlantic bluefin tunamanagement units.

The second set of bootstrap analyses evaluated the non-uniform number of samples per size class in the compiled dataset. In this bootstrap, the model was forced to have an equal number of samples for each 5 cm bin size for the whole range of the data (30–300 cm SFL). In this case the model fits favored the under-represented smaller fish and failed to fit larger sized fish. Fitting diagnostics indicated a poor fit, with heavy trends of residual patterns, and greater AIC and RME values compared to the base models (Fig 8).

**Fig 9:**
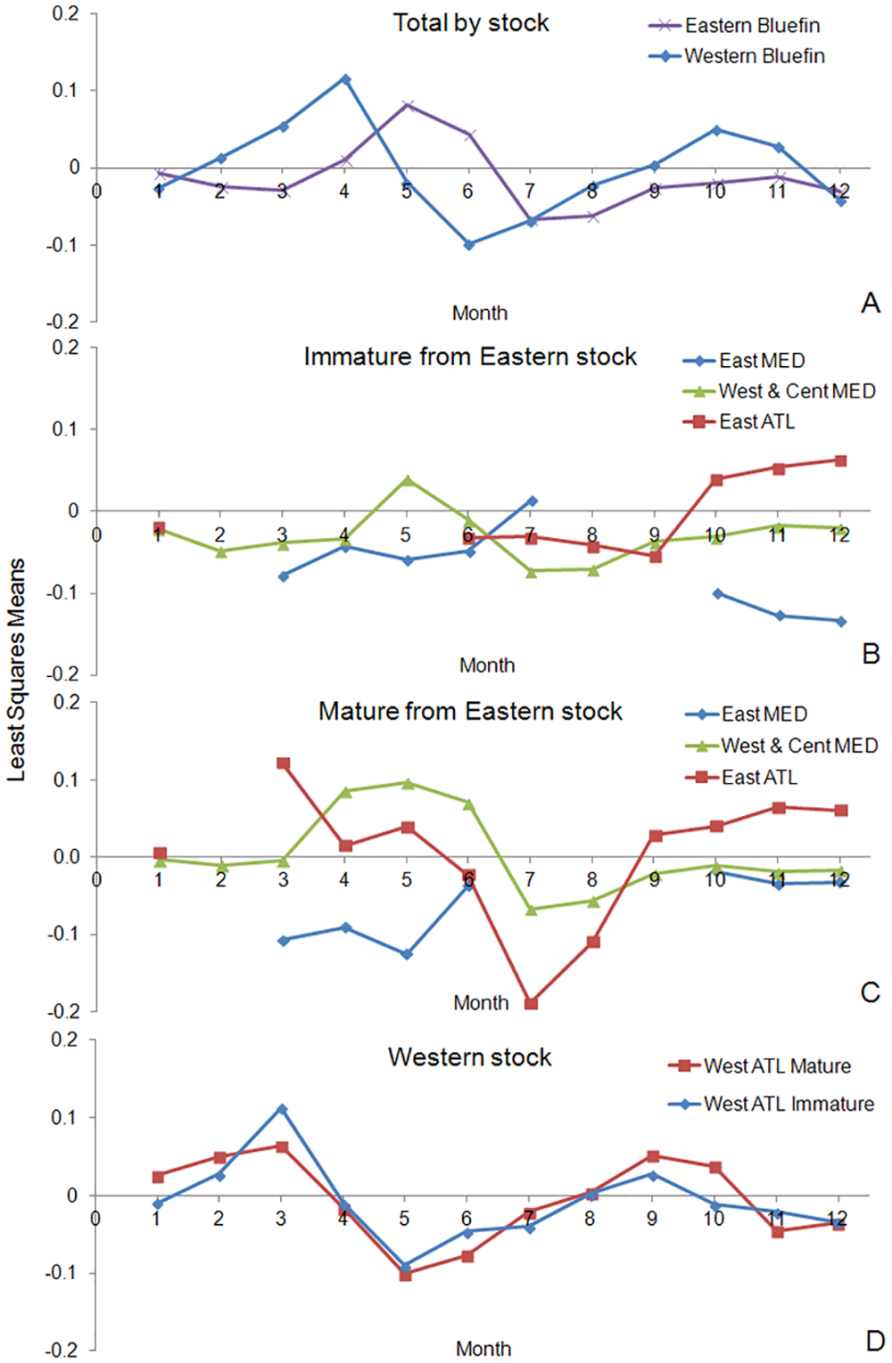
Monthly residual trends for WLRs for Atlantic bluefin tuna. A) by stock, B&C) when including maturity and area factors for Eastern and D) when including maturity factor for Western bluefin.

In the analysis yearly variations of fish condition were statistically significant, in part due to the large number of observations in the models. However the deviance explained by the monthly effect was much higher than for the yearly effect. The ratios of deviance explained by month over year were 3.9 and 3.5 for Eastern and Western bluefin, respectively. Given that the objective was to estimate a function that can be applied multi-yearly, the factor year was not included in the final model. The analyses by month indicated that for Eastern bluefin the fish were heavier in May-June and lighter in July-August (Table 4, Fig 9A). Compared to the average annual mean weight, the seasonal variations ranged from minus 5% to plus 4% for Eastern bluefin, and from minus 9% to plus 7% for Western fish, with the absolute variations in weight greater for larger fish. For Western bluefin the seasonal effect appeared greater, and offset compared to the East. Fish were heavier in March-April and lighter in June-July, with a second peak of heavier weight in October, followed by declines in December-January (Fig 9A).

**Table 4:** Estimated coefficients alpha and beta for the monthly weight-length relationship for Atlantic bluefin tuna ($\text{RWT}\_\text{st}{\text{d}}_{\text{M}}=\text{alpha}\times \;\text{SFL}\_\text{st}{\text{d}}^{\left(\text{bet}{\text{a}}_{\text{lsMonth}}\right)}$). All functions correspond to straight fork length (SFL) in centimeters and round weight (RWT) in kilograms.

Month	Western stock		Eastern stock	
	alpha	beta_lsMonth_	alpha	beta_lsMonth_
January	1.771E-05	2.99789075	3.508E-05	2.87767028
February	1.771E-05	3.00292688	3.508E-05	2.87585971
March	1.771E-05	3.00833823	3.508E-05	2.87549667
April	1.771E-05	3.01618530	3.508E-05	2.87961024
May	1.771E-05	2.99883106	3.508E-05	2.88691388
June	1.771E-05	2.98858373	3.508E-05	2.88309179
July	1.771E-05	2.99247363	3.508E-05	2.87153307
August	1.771E-05	2.99823761	3.508E-05	2.87200195
September	1.771E-05	3.00172394	3.508E-05	2.87577309
October	1.771E-05	3.00774859	3.508E-05	2.87633165
November	1.771E-05	3.00493806	3.508E-05	2.87716283
December	1.771E-05	2.99596985	3.508E-05	2.87529487

Monthly trends in the weight at size residuals when including maturity and geographical area factors (east) or only maturity (west) indicated statistical significance of the area and maturity factors. For the Eastern stock (Fig 9B and 9C), the analyses indicated greater variance in weight at size for mature fish, even more so for the eastern Atlantic compared to the Mediterranean Sea. Eastern Atlantic mature fish were in good body condition in spring and autumn, and in low condition in summer months; immature fish showed a gain in somatic condition in the autumn. There were differences in the seasonal changes of weight between the west-central and the eastern Mediterranean. In the west-central area, mature fish had larger than expected weights during the months of April through June, while mature fish had lower than expected weights at size for the July-August months. Immature fish exhibited the same trends, but only showed higher than expected weights at size in May. In contrast, mature fish in the eastern Mediterranean showed poor condition between March and June, and despite a slight recovery thereafter, continued to remain below the expected weight into autumn. It should be noted that sampling for this area was incomplete, with few years and months sampled. For the Western bluefin stock (Fig 9D), the sampling was limited in the Gulf of Mexico, with most samples from the north-western Atlantic coast. For mature Western bluefin, heavier weights at size were prevalent early in the year, peaking in March, followed by lighter fish in May through June, and finally recovering at the end of summer and into the autumn. Immature Western bluefin followed a similar pattern with good condition at the end of the winter months, poor condition from May to June and recovering weight in September.

## Discussion

This study provides robust biometric relationships for Atlantic bluefin tuna based on extensive sampling covering most of the fishing areas for this species in the North Atlantic Ocean and Mediterranean Sea. Measurements from over 140 thousand sampled fish were compiled, providing samples with sufficient range for both length and weight conversion factors. However, because of the diverse types of length and weight measurements obtained from different fisheries, we pooled the data by stock and standardized to a common measurement type of weight (round weight) and length (straight fork length) for the weight-length equations. This allowed for the combination of data across multiple areas and fisheries, substantially improving the sampling.

Even though the present database was compiled from extensive sampling carried out over 15 years, all months, geographic areas and fishing gears, it is still an unbalanced sampling dataset. This was caused by the majority of the data coming from fishery dependent operations which are strongly influenced by the bluefin biology and fishing regulations. The strong seasonality in the bluefin tuna fisheries, which mostly capture a limited size range, is a consequence of the migratory behavior and the wide distribution area of this species, in which juveniles have different migratory patterns than adults [24, 26]. In addition, management regulations such as time-area closures and minimum sizes [15] have restricted data collection. These factors complicate biometric studies from catches, but fishery-independent samples rarely exist for bluefin tuna, largely due to the absence of scientific surveys for the species despite its high market demand and value.

The representativeness of the actual sampling compared to the simulated population indicated that available size data for small bluefin is truncated for fish smaller than 75 and 175 cm SFL for the Eastern and Western stocks respectively; a fact clearly attributed to minimum size restrictions. However, there is also a dip in the distribution of the samples for Eastern bluefin between the sizes ranges 130 to 180 cm SFL (Fig 4, top panel). This feature could be explained by strong size selectivity of the major bluefin fisheries in the Mediterranean Sea, but could also be a function of the availability of these size ranges to the fisheries. In fact, the same reduced sampling for medium fish (130–180 cm SFL) was achieved on the study from Baglin [27] for Western bluefin size relationships. Recently it has been indicated that mature bluefin may skip spawning due to unfavorable body condition, therefore bypassing migration to spawning sites [28, 29], which could explain non-availability of these size classes to the main fisheries. Nevertheless, based on the population simulation analyses, the overall sampling and size coverage for both bluefin stocks is quite good and close to a hypothetical population, considering the minimum size/weight regulations. Though, in some cases, the proportion of large fish samples (>200 and >250 cm SFL for Eastern and Western bluefin, respectively) is higher than the expected from the simulated population.

A particular feature of the observed weight size data is the large variability of weight at length, particularly in the intermediate sizes (90–120 SFL cm for Eastern and 130–180 SFL cm for Western bluefin) where CVs peaked over 30%, compared to the rest of the sizes where CVs ranged from 10 to 20%. This increase in variance is likely associated with the onset of maturity and the physiological changes associated with spawning. However, when assuming that fish weight at size commonly follows a power function type, such large intermediate variance would predict even greater variability in weight for the larger fish, which is simply improbable from a biological point of view. Therefore, the final weight at size model fit by stock used a weighted factor (the inverse observed CV by 5 cm SFL size class) to avoid undue influence of extremely variable weights at sizes upon the model fit.

Seasonality in the weight-length ratio has been previously documented in bluefin, with juveniles and adults growing rapidly during the summer and early autumn, and negligible growth in the winter [4, 24, 26]. Seasonal variability in condition for bluefin has been modeled considering the metabolic costs during migration and spawning in relation to foraging opportunities [30]. The extensive sampling of present data allowed characterization of seasonal changes in condition; especially for adults, as monthly variations of fish condition are expected to be lower for juveniles (Fig 9). The monthly residual trends of weight at size for mature adults is likely associated with spawning costs and feeding immediately following return from the spawning grounds. Declines in condition coincided with the spawning season and locations (April to June, Gulf of Mexico and eastern Mediterranean; May-July, western and central Mediterranean [24, 31]). The loss of body fat during the spawning season in response to its reproductive effort has been referenced [30, 32], as well as the increase in condition of large bluefin from summer to the beginning of autumn when they return to feeding areas [26, 30, 33, 34]. Rodriguez-Roda [35] also observed that adult fish lost about 15% of their weight between the pre and post spawning state from May to August (specimens migrating through the Strait of Gibraltar towards the spawning areas in the western Mediterranean and post spawning tuna performing the reverse trophic migration towards the Atlantic) in the traps along the south Atlantic coast of Spain. This correlation of fish condition and month was not so clearly observed in the eastern Mediterranean area (Fig 9C), likely due to low sample coverage in this area. However, the lowest annual condition was observed in the month of May, coinciding with the peak spawning in this area of the Mediterranean. Seasonal analysis also showed a great variation in the condition of sexually mature bluefin sampled in the east Atlantic (outside the Mediterranean spawning ground) with a weight loss in July and August, and a recovery in stores (and weight) lost during migration and spawning when arriving at feeding grounds in the Atlantic at the end of summer. It can be argued that the major geographical areas used in the seasonal fish condition analysis (western and eastern Atlantic, west-central and eastern Mediterranean) are too extensive. However, given the speed and scale of migration undertaken by Bluefin Tuna at different stages of their life [24], the use of smaller geographical areas would not sufficiently cover the whole range of sizes throughout the entire year. West Atlantic mature bluefin are one example of this, as they remain in the Gulf of Mexico during their spawning season from February to June [5]. Seasonal variability in condition from this study corroborates the convenience of having separated seasonal WLRs by stock due to differences in weight gains and losses related to spawning and feeding behavior.

While some meristic equations estimated in this study were similar to the ones currently used by ICCAT, others show substantial divergence. The new equations have the benefit of greater spatial and temporal sampling coverage and full documentation of the sample origin and metadata (fishery, date, sample size, etc). The length-length conversions factors for straight versus curved fork length (SFL-CFL) from this study are almost identical for both stocks and to the function used by ICCAT [34]. The weight-weight conversion factor obtained for round versus dressed weight (RWT-DWT) was compared to the one used in ICCAT (RWT = 1.25*DWT; [36]). Our results for Western bluefin for RWT-DWT coincide with the ICCAT factor, except for fish less than 100 kg RWT, a size range which our sampling does not cover. The present function for the gutted and gilled-round weight relationship differs from ICCAT functions. The factor 1.16 cited in [36] and the factor 1.13 for the Mediterranean [37] showed up to 15% divergence as size increased, when compared to estimates from this study for the Eastern stock. Both ICCAT functions are cited without any accompanying information on the sampling.

Numerous WLRs have been presented for this species since the mid-20th century [33, 35, 38], most of which consist of samples from limited geographical areas and single fisheries. Moreover, many of these were based on data from specimens sampled only in the Mediterranean and not the entire Atlantic (for a review see [10, 39]). The WLRs obtained in this study differ from the equations currently used in ICCAT. The final model for the Eastern bluefin stock differs slightly from the one of Rey and Cort (unpublished, collected in [40]), estimating between 5% to 10% higher weight by length (Fig 5). The equation presented by the latter authors has been used in previous ICCAT bluefin assessments for fish less than 101 cm SFL. The biggest difference was found between this study and the equation from Arena (unpublished, collected in [40]) WLR which predicted 11% -17% greater weights at length for fish above 200 cm SFL (Fig 5).

This is important because the current WLR used in stock assessments [15] for Mediterranean fish > 100 cm SFL is one attributed to Arena (unpublished, collected in [40]). This Arena function overestimates weight compared with the current study and those of other Mediterranean functions previously reported [39, 41–44]. Furthermore, the Arena WLR overestimated weights from farmed tuna [13, 45, 46], even after one year ranching which is surprising as reared tuna is known for their fat condition. Unfortunately the original data used to fit the Arena (unpublished, collected in [40]) WLR could not be found, however one citation [47] provides a table of 1 cm binned length and weight measurements which seem to likely represent the data used to fit the relationship. The data in this table exhibit no variability in weight at length, continually increase in weight at length and almost exactly match the Arena (unpublished) WLR indicating that these data likely were subject to some filtering or averaging prior to fitting the curve and are unlikely to represent the raw observations. Furthermore, the data used by Arena et al. [47] came from traps and purse seiners in May-July from the southern Tyrrhenian Sea; a time and location where fish were likely to be at the peak of spawning and unlikely to represent the whole population.

This study results for the western Atlantic WLR showed lower weight at length compared with the annual and September functions from Parrack and Phares [34], with differences of 10% and 11% respectively (Fig 5). Busawon et al. [11] described an overestimation of large bluefin weight when using the Parrack and Phares [34] monthly functions, especially in September. This is also relevant since September Parrack and Phares [34] function has been used for Western bluefin stock assessment [15].

The WLRs developed in this study differ from many used in the most recent 2014 assessment of Atlantic bluefin tuna. In particular, the current study WLRs indicate a smaller weight at length than the Arena (unpublished, collected in [40]) and Parrack and Phares (1979) September relationships used in the previous stock assessments for Eastern and Western bluefin, respectively. However, both Arena (unpublished, collected in [40]) and Parrack and Phares [34] share very limited or no information about sampling and the type of data filtering applied, and represent only a restricted time and area strata. Therefore, the suitability of these functions to represent the whole population is questionable and recent studies indicate the possibility that they may overestimate fish weight [10, 11].

This study compiles the most comprehensive set of biometric data available for Atlantic bluefin tuna. The relationships presented here represent state-of-the-art model fitting and provide updated information which is critical for the assessment and management of Atlantic bluefin tuna. The annual relationships will be of value for assessments that work on population averages, while the monthly relationships reflect the seasonal life history variation related to feeding and spawning, and may be of use when specific WLRs are necessary. Nonetheless these relationships do not necessarily reflect local fishery conditions which may differ from the population averages proposed herein.

Research on biometric relationships is not currently considered an interesting science by fisheries scientists [48], but conversion factors need to be used in fisheries research and are essential in data processing in all assessments of fish stocks. Our results affect the current estimates of weight used in the bluefin stock assessment and can contribute to improving the estimation of the weight gain of fattened bluefin in cages. The impact of using these new WLRs must be carefully evaluated as they may have a substantive influence on the results, depending upon where they are used in the stock assessment.

## Supporting Information

S1 Fig Number of collected and standardized weigh-length samples (RWT-SFL pairs of values) per 5 cm SFL size bins by major geographical areas , A) east Atlantic, B) western and central Mediterranean, C) eastern Mediterranean and D) western Atlantic.
(TIF)
