# Correction: Atlantic Bluefin Tuna (*Thunnus thynnus*) Biometrics and Condition

**DOI:** 10.1371/journal.pone.0157291

**Published:** 2016-06-06

**Authors:** Enrique Rodriguez-Marin, Mauricio Ortiz, José María Ortiz de Urbina, Pablo Quelle, John Walter, Noureddine Abid, Piero Addis, Enrique Alot, Irene Andrushchenko, Simeon Deguara, Antonio Di Natale, Mark Gatt, Walter Golet, Saadet Karakulak, Ai Kimoto, David Macias, Samar Saber, Miguel Neves Santos, Rafik Zarrad

Table 2 was published with an incorrect number of specimens (n) and coefficient of determination (r^2^) for the weight conversion factors RWT-GGWT and GGWT-RWT for the East and Mediterranean stock unit. As the correct r^2^ is lower than the high coefficient of determination chosen as threshold for using the biometric relationship (*r*-square ≥ 0.98), we prefer to delete both relationships (GGWT = alpha + beta*RWT; and RWT = alpha + beta*GGWT) in Table 2. The only change implies deleting rows 11 and 12 from Table 2.

The corrected Table 2 is the following:

**Table 2:** Atlantic bluefin tuna biometric relationships for the Eastern Atlantic and Mediterranean stock. Independent and dependent variables (X and Y), number of specimens (n), parameters of the linear and nonlinear equations and coefficient of determination (r^2^). Straight fork length (SFL), curved fork length (CFL), straight first dorsal fin length (LD1), head length (HeadL), preopercular length (PreopL), round weight (RWT), gutted weight (GWT), gutted and gilled weight (GGWT), gutted, gilled and tailed weight (GGTWT) and dressed weight (DWT). Method A: Fit Robust Estimate; B: Nonlinear fit CV weighted; C: Nonlinear fit Gauss-Newton. Standardized WLRs (RWT_std-SFL_std). Length in centimeters and weight in kilograms.

Function relationship by stock / East stock unit	X	Y	X range	Y range	n	Months sampled	alpha	beta	r^2^	Residual standard error	Method
**Length conversion factors**:											
LD1 = alpha + beta*SFL	SFL	LD1	56–300	17–71	636	2–8, 10, 11	5.6891	0.2543	0.978	2.052	A
CFL = alpha + beta*SFL	SFL	CFL	78–242	84–252	222	6–7	-1.887	1.0507	0.990	4.121	A
SFL = alpha + beta*LD1	LD1	SFL	17–71	56–300	636	2–8, 10, 11	-19.733	3.8648	0.978	8.063	A
CFL = alpha + beta*LD1	LD1	CFL	24–71	84–283	312	5–7	-27.832	4.1273	0.964	8.839	A
LD1 = alpha + beta*CFL	CFL	LD1	84–283	24–71	312	5–7	7.9182	0.2355	0.964	2.116	A
SFL = alpha + beta*CFL	CFL	SFL	84–252	78–242	222	6–7	2.9457	0.9442	0.990	3.886	A
HeadL = alpha + beta*CFL	CFL	HeadL	84–284	22–74	306	5, 7	4.4041	0.2242	0.865	3.048	A
PreOP = alpha + beta*CFL	CFL	PreOP	153–284	33–74	294	5	1.0934	0.1892	0.646	3.100	A
PreOP = alpha + beta*HeadL	HeadL	PreOP	38–74	33–74	294	5	-2.2179	0.8358	0.783	2.428	A
**Weight conversion factors**:											
GWT = alpha + beta*RWT	RWT	GWT	0.3–370	0.3–358	236	5–11	-0.2169	0.9540	1.000	1.090	A
RWT = alpha + beta*GWT	GWT	RWT	0.3–358	0.3–370	236	5–11	0.2312	1.0479	1.000	1.140	A
**Weight—length relations**											
RWT_std = alpha*SFL_std^beta	SFL	RWT	27–300	0.25–513	74272	1–12	3.51E-05	2.8785	na	15.965	B
GGTWT = alpha*SFL^beta	SFL	GGTWT	75–281	8–362	8034	1, 8–12	4.59E-05	2.8077	na	13.407	C
GGWT = alpha*SFL^beta	SFL	GGWT	55–289	2.8–385	3469	1–12	1.07E-04	2.6301	na	14.249	C
GGWT = alpha*CFL^beta	CFL	GGWT	94–289	10–338	4962	4–8	2.55E-05	2.8938	na	15.357	C
GGWT = alpha*LD1^beta	LD1	GGWT	29–76	20–350	2044	5–7, 9	3.85E-03	2.6211	na	21.820	C
RWT = alpha*LD1^beta	LD1	RWT	17–79	3–425	2796	2–8, 10–11	1.12E-03	2.9180	na	20.019	C

The last two sentences of the second paragraph of the Discussion section are incorrect. They should read:

We could not provide an updated gutted and gilled-round weight relationship (r-square <0.98), therefore, current ICCAT factors of 1.16 for the Atlantic [36] and the factor 1.13 for the Mediterranean [37] are the only available conversion factors, although both are cited without any accompanying information on the sampling.
