# Supplementary figures and images for: Atlantic Bluefin Tuna (Thunnus thynnus) Biometrics and Condition
Source: PLoS One. 2015 Oct 27;10(10):e0141478. doi: 10.1371/journal.pone.0141478 (PMC4623980; doi:10.1371/journal.pone.0141478)

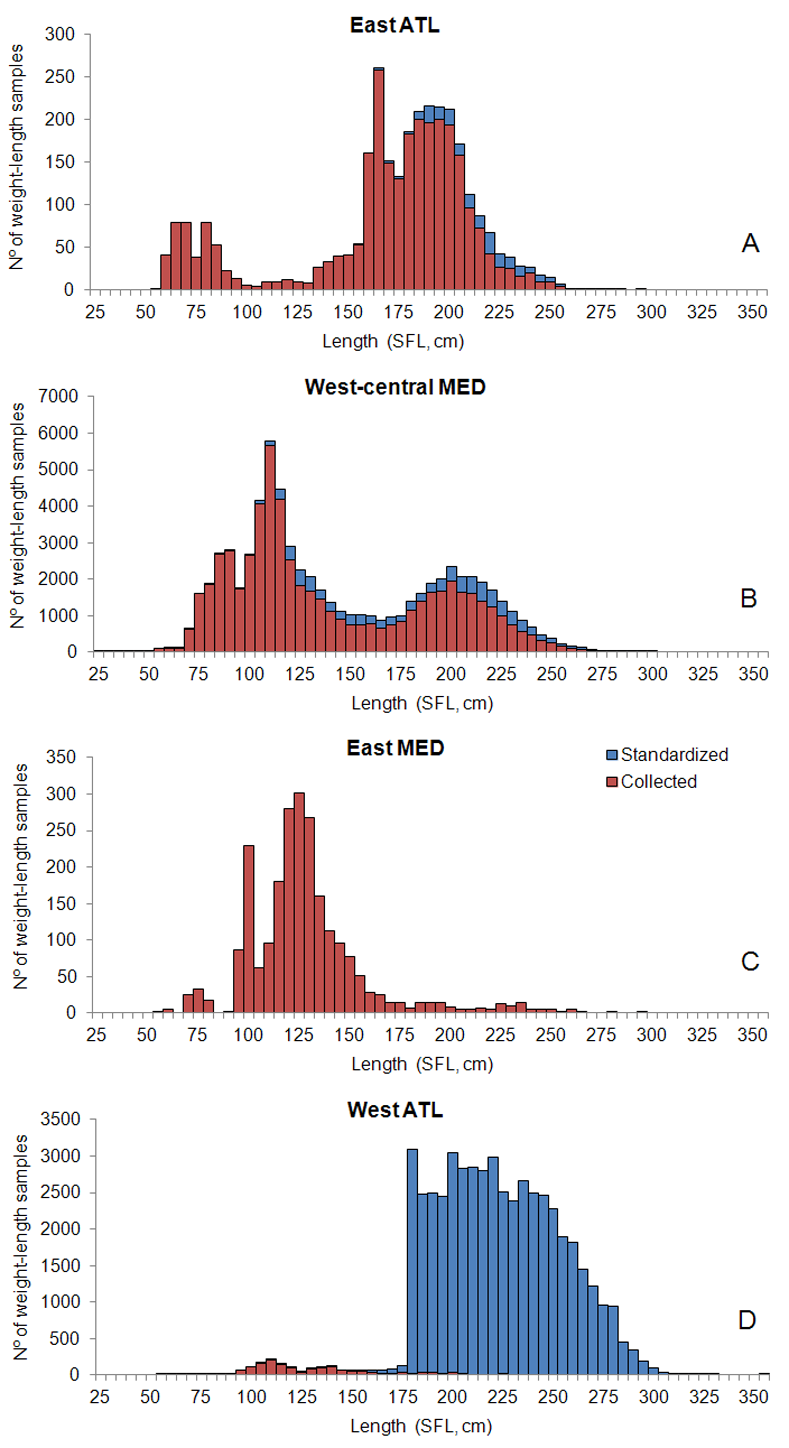

Supplement: S1 Fig — , A) east Atlantic, B) western and central Mediterranean, C) eastern Mediterranean and D) western Atlantic. (TIF) [file pone.0141478.s001.tif]
